# UV induced visual cues in grasses

**DOI:** 10.1038/srep02738

**Published:** 2013-09-24

**Authors:** Sabulal Baby, Anil John Johnson, Balaji Govindan, Sujith Lukose, Bhaskaran Gopakumar, Konnath Chacko Koshy

**Affiliations:** 1 Phytochemistry and Phytopharmacology Division, Jawaharlal Nehru Tropical Botanic Garden and Research Institute, Pacha-Palode, Thiruvananthapuram 695562, Kerala, India; 2 Plant Genetic Resources Division, Jawaharlal Nehru Tropical Botanic Garden and Research Institute, Pacha-Palode, Thiruvananthapuram 695562, Kerala, India; 3 These authors contributed equally to this work.

## Abstract

Grasses are traditionally considered as wind pollinated, however, field observations confirmed frequent insect visits to grass flowers, suggesting insect pollination. Fruit and seed predators inflict heavy losses to cereals and millets during their growth, maturation and storage. The actual factors guiding insects and predators to grass flowers, fruits and seeds are not clear. Here, we report attractive blue fluorescence emissions on grass floral parts such as glumes, lemma, palea, lodicules, staminal filaments, pollens and fruits in ultraviolet (UV) 366 nm, whereas the stigmatic portions were not blue, but red fluorescent. We characterized the blue fluorescent constituent in grass reproductive structures as ferulic acid (FA). Fluorescence spectra of blue-emitting grass floral, seed extracts and isolated FA on excitation at 366 nm showed their emissions at 420–460 nm. We propose these FA-based blue fluorescence emissions in grass reproductive structures as visual cues that attract pollinators, predators and even pests towards them.

Grasses of monocot family *Poaceae* include cereals (rice, wheat, maize), millets (bajra, ragi), bamboos and fodders. They are vital food sources for humans and animals^1^. The lignocellulosic biomass of grasses is considered as a renewable feedstock for biofuels^2^. Though grasses are known to be wind pollinated, recent studies reported bee and insect visits to bamboo flowers in south America^3^, south India^4^,^5^ and central China^6^. Moreover, insect visits to grass flowers (florets) were observed in habitats where wind is negligible^3^. Our own field observations on rice, maize and bamboos^4^,^5^ and other reports^7^,^8^,^9^ provide sufficient documentation of bee, insect and pest visitations to grasses at flowering and fruiting stages. These reports led to strong suspicion that floral visitors might play a role in pollination. Grass flowers are small but numerous in clusters, and are not showy compared to orchids or roses. With no attractive colour, nectar or scent, and pollen as the only reward^3^, it is not clear how bees and insects are enticed to grass flowers. Similarly, rodents and other animals inflict heavy loses to grass fruits and seeds (food grains), and the factors guiding these predators to them are unclear^10^,^11^. Here, we show that FA-based attractive blue fluorescence emissions from grass flowers, fruits and seeds play a potential role in insect/animal attraction.

## Results

**Figure 1:**
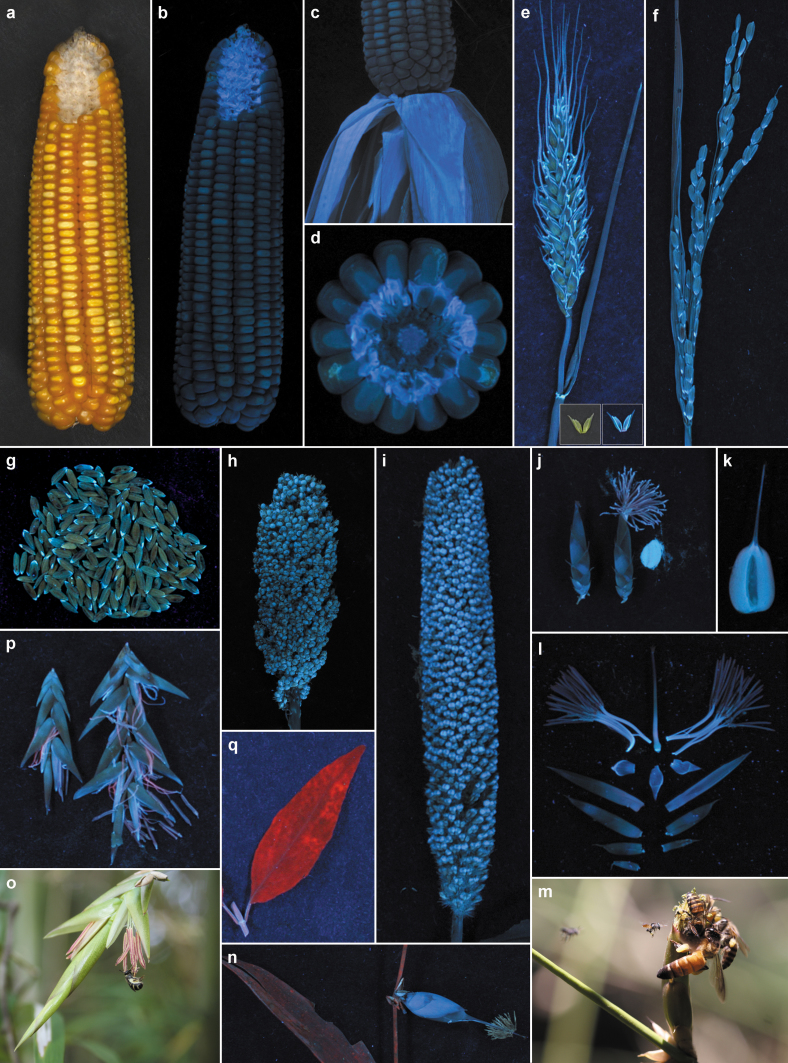
Blue fluorescence emission patterns in grasses at UV 366 nm, bee activity in field conditions. (a) Maize cob, grains removed portion exposing floral parts, in white light. (b) Same at 366 nm. (c) Maize cob with bracts, 366 nm. (d) Maize cob cross section showing blue fluorescent floral parts and grains, 366 nm. (e) Wheat ear with leaf at UV 366 nm, its lemma and palea in white light (inset, left) and at 366 nm (inset, right). (f) Rice ear with leaf at 366 nm, with its glowing glumes. (g) Rice grains, with their glowing glumes, 366 nm. (h) *Sorghum bicolor* ear with mature fruits, 366 nm. (i) *Pennisetum glaucum* ear with immature fruits, 366 nm. (j) *Ochlandra travancorica* spikelet at 366 nm, female stage showing stigma (left), male stage showing exserted stamens (middle) and pollen grains (right). (k) *O. travancorica* fruit longitudinal section, 366 nm. (l) *O. travancorica* floral parts at 366 nm, glumes (lower 5), lemma (above glumes, left), palea (above glumes, right and opposite to lemma), three lodicules (above lemma and palea), staminal filaments and anthers (either side of pistil), pistil (female part) showing ovary (bottom bulged part), style (middle part) and stigma (tip). (m) Bee activity on *O. travancorica* spikelet (male stage) in the field, in daylight, *Apis dorsata* (largest), *A. cerena indica* (medium), *Trigona iridipennis* (small on flight) foraging on anthers. (n) *O. travancorica* fruit and its leaf, 366 nm. (o) Bee activity on *Bambusa pallida* spikelet in the field, in daylight, *Halictus taprobane* foraging on anthers. (p) *B. pallida* spikelet showing opened florets, 366 nm. (q) *Clinacanthus nutans* (dicot) leaf, 366 nm. (Plant specimens collected by K. C. K., B. G.^2^ and S. L.; ultraviolet scans taken by S. B., A. J. J. and B. G.^1^; bee activities photographed by K. C. K. and B. G.^2^).

We observed strong blue fluorescence emission patterns from the glumes, lemma, palea and lodicules of grasses *Oryza sativa* L. (rice), *O. rufipogon* Griff. (red rice), *Triticum aestivum* L. (wheat), *Zea mays* L. (maize), *Sorghum bicolor* (L.) Moench (sorghum, ‘cholam'), *Sorghum bicolor* (L.) Moench (sorghum, ‘kattucholam'), *Eleusine coracana* (L.) Gaertn. (ragi, finger millet), *Pennisetum glaucum* (L.) R. Br. (bajra), *Ochlandra travancorica* (Bedd.) Gamble, *Bambusa pallida* Munro and *Melocanna baccifera* (Roxb.) Kurz, both in flowering and fruiting stages at UV 366 nm (Fig. 1b–j, l, n, p). Lodicules, with only known role in opening of florets, were strongly blue fluorescent (Fig. 1l). Staminal filaments were intensely blue fluorescent in *O. travancorica*, *B. pallida* and *M. baccifera* (Fig. 1l), but relatively less fluorescent in *O. sativa*, *O. rufipogon* and *T. aestivum*. Pollen grains of *O. travancorica*, *B. pallida* and *M. baccifera* were strongly blue fluorescent (Fig. 1j). When fluorescent pollens were dusted off, their anthers appeared mild red with chlorophyll emissions (Fig. 1j, l, p). Anthers in *O. sativa*, *O. rufipogon*, *T. aestivum*, *Z. mays*, *E. coracana* and *P. glaucum* appeared faintly blue fluorescent. Pollens in these tiny grass florets were relatively less fluorescent. Ovaries and styles in all grass species observed were blue fluorescent (Fig. 1l). Stigmas were not blue fluorescent but mildly red (Fig. 1l).

Unlike in other flowering plants, the floral parts in grasses such as glumes, lemma and palea do not fall after pollination, but remain as persistent protective structures in mature fruits (Fig. 1n). Glumes in *O. sativa* and *O. rufipogon* grains emitted intense blue fluorescence at UV 366 nm (Fig. 1f, g). Glumes of aborted spikelets were relatively less blue fluorescent compared to fruiting ones. In *O. travancorica* fruits, persistent lemma and palea were blue fluorescent at the outer surface and their inner unexposed surfaces were less fluorescent (Fig. 1n). Both fruit outer cover and endosperms of these grasses were fluorescent, with inner (cut or crushed) portions showing significantly higher intensities of blue emissions (Fig. 1d, k). In *Z. mays*, the sheaths covering its cobs were strongly blue fluorescent with highest emissions from innermost sheaths (Fig. 1c). Common fodder grasses *Pennisetum polystachion* (L.) Schult. and *Axonopus compressus* (Sw.) P. Beauv. also showed similar blue fluorescence patterns on their relatively tiny floral and fruiting structures.

**Figure 2:**
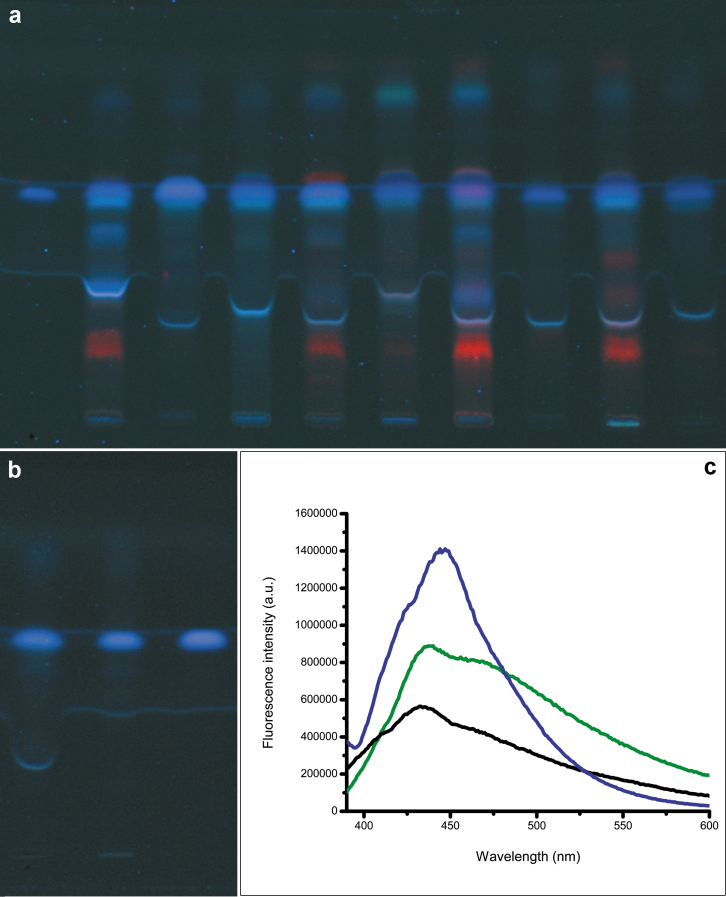
HPTLC profiles of blue and red metabolites in the leaves and reproductive structures of grasses at UV 366 nm, fluorescence emission spectra. (a) Tracks (left to right) (t1) FA standard, (t2) *Zea mays* leaves, (t3) *Z. mays* internal bracts, (t4) *Z. mays* male flower, (t5) *Oryza sativa* leaves, (t6) *O. sativa* spikelet, (t7) *Ochlandra travancorica* leaves, (t8) *O. travancorica* spikelet, (t9) *Bambusa pallida* leaves and (t10) *B. pallida* spikelet. (b) Tracks (left to right) (t1) *T. aestivum* seed extract, (t2) FA (isolated) and (t3) FA (standard). (c) Fluorescence emission spectra of *T. aestivum* seed extract (green), isolated FA (black) and standard FA (blue) at an excitation λ 366 nm.

We isolated the blue fluorescent fractions from fresh grass specimens viz. *Z. mays* (leaves, male flowers, external bracts, internal bracts, lemma/palea/lodicule, immature seeds), *O. sativa* (leaves, spikelets, immature seeds), *E. coracana* (mature seeds), *O. travancorica* (leaves, spikelets, lemma/palea/staminal filaments/pollen) and *B. pallida* (leaves, spikelets, lemma/palea/lodicules, immature seeds) by alkaline hydrolysis and extraction (see Methods). Blue fluorescent fractions were also isolated from mature seeds (dry, stored) of *Z. mays*, *O. sativa*, *T. aestivum* and *B. pallida*. On HPTLC profiling at 366 nm, *Z. mays*, *T. aestivum*, *O. sativa*, *E. coracana*, *O. travancorica* and *B. pallida* extracts showed blue spots at retention factor (R*f*) 0.54, exactly matching with FA (Fig. 2a, b). FA (blue) was the major spot in extracts of floral parts and seeds of these grasses. Leaf extracts of grasses showed multiple chlorophyll (red) signals in addition to FA (Fig. 2a). FA contents in leaves, floral parts and seeds of *Z. mays*, *O. sativa*, *T. aestivum*, *O. travancorica* and *B. pallida*, estimated by alkaline hydrolysis and HPTLC-densitometry (see Methods), are given in Table 1. For comparison with dicot plants, *Clinacanthus nutans* (Burm.f.) Lindau (*Acanthaceae*) and *Synedrella nodiflora* (L.) Gaertn. (*Compositae*), were randomly selected and their fresh leaves were extracted by the same procedure. *C. nutans* and *S. nodiflora* leaf extracts showed strong red chlorophyll signals at 366 nm, but the blue-emitting FA signals detected were very feeble (Fig. 1q, S1).

Blue FA spot was isolated in pure form from *T. aestivum* seed extract (see Methods) (Fig. 2b). On excitation at λ 366 nm, *T. aestivum* seed extract, isolated FA and standard FA showed fluorescence emission maxima in the blue (420–460 nm) region (Fig. 2c). But, *C. nutans* leaf extract showed strong red chlorophyll emissions at 655–695 nm. Both isolated and standard FAs showed overlapping UV absorption spectra with their maxima at 322 nm.

## Discussion

We found grass inflorescences very attractive with UV-induced blue emission patterns. These blue emissions from critical floral parts of grasses are not known so far. UV induced floral emission patterns were reported as strong visual signals to pollinators in colourful dicot flowers of *Hypericum calycinum* (flavonoids)^12^ and *Mirabilis jalapa* (betaxantin, betanin)^13^. Recently, we reported strong blue fluorescence emissions from prey traps of carnivorous plants *Nepenthes*, *Sarracenia* and *Dionea* in UV^14^. Over a decade ago, we recorded bee (*Apis*, *Halictus*, *Trigona*, *Braunsapis*, *Ceratina*) visits to six woody bamboos (*Ochlandra travancorica*, *O. ebracteata*, *O. scriptoria*, *Bambusa bambos*, *B. vulgaris*, *Bambusa* sp.) in the same geographical fields in south India as described in this study^4^,^5^. Since then we observed similar bee visit patterns to the bamboos in our Institute Bambusetum (N 08° 45.268′–434′ E 077° 01.429′–594′)^15^. Our recent observations clearly demonstrate bee visits (and foraging) on bamboos (Fig. 1m, o). These bees were netted and identified as *Apis dorsata* Fabricius (largest), *A. cerana indica* Fabricius (medium) and *Trigona iridipennis* Smith (small, in flight) on *O. travancorica* (Fig. 1m) and *Halictus taprobane* Cameron on *B. pallida* (Fig. 1o). Our data (Fig. 1m, o)^4^,^5^ and other previous studies^3^,^6^ found bees and other visitors only on the male stages of bamboo flowers, and reported pollen collections by them. These visitor activities were not observed in the female stages of their flowers^3^,^4^,^5^,^6^. Blue signaling from the male stages of flowers and pollens observed in this study (Fig. 1j, l, p) is coinciding with the visitor hits and pollen collection patterns (Fig. 1m, o) reported earlier^3^,^4^,^5^,^6^.

We also observed distinct blue emissions at UV 366 nm from the endosperms of the cereals, millets and bamboos (Fig. 1d, k). Seed predators or dispensers like birds, rats and other small mammals could see these UV induced blue fluorescence emissions^16^,^17^,^18^,^19^,^20^,^21^,^22^,^23^. Rats are known fruit predators of bamboos^10^,^11^. Rodent and other predation losses of cereals and millets in seedling, grain and warehouse stages are hugely significant^11^. Most nocturnal species, active in late evenings and in the darkness of the night, have adapted sensitive vision aiding them to find their food and mates^17^,^19^,^20^,^21^,^22^,^23^,^24^. Even low levels of UV in nocturnal conditions could lead to blue emissions from grass flowers, fruits and seeds. These fluorescence emissions from grass reproductive structures are in the blue region of the visible spectrum, which is the best detectable range for arthropods, birds, rats and other small mammals^16^,^17^,^18^,^19^,^20^,^21^,^22^,^23^. These blue emissions from grass flowers and seeds could attract seed dispensers, predators and even insect pests towards them^18^,^20^. Under UV, the reproductive structures of grasses are truly ‘showy'.

Our UV induced fluorescence emission (Fig. 1), HPTLC (Fig. 2a, b) and fluorescence spectral (Fig. 2c) data clearly showed that FA is causing the blue emissions from the floral parts, fruits and seeds of grasses. The role of cell wall bound FA as distinct visual cues (blue emissions) in vital reproductive structures of grasses is not known so far. On estimation, in maize and rice, FA contents in floral portions were higher compared to their leaves, up to 3.23 times in internal bracts of maize cob compared to its leaves (maize, male flowers:leaves 2.34, cob external bracts:leaves 1.77; rice, spikelets:leaves 1.42, immature seeds:leaves 1.16) (Table 1). Dry seeds of both maize (seeds:leaves 0.80) and rice (seeds:leaves 0.06) showed relatively lower FA contents compared to their fresh leaves (Table 1). Previous studies found graminaceous cell walls as rich natural sources of FA (up to 3.0%, dr. wt. in maize bran), and it is mostly bound in the form of ferulate-polysaccharide-lignin complexes^25^,^26^,^27^,^28^. FA contents in rice grains^29^ and rice endosperm cell walls^28^,^30^,^31^ were estimated as 0.0061–0.0362 (%, dr. wt.) and 0.91 (%, dr. wt.), respectively. In bamboos, floral portions of *O. travancorica* (spikelets:leaves 0.44, flowers:leaves 0.28) and *B. pallida* (spikelets:leaves 0.68) showed a lower content of FA compared to their leaves (Table 1). Bamboo flowers are relatively big, and FA-based emissions are highest in their staminal filaments and tiny lodicules (Fig. 1l). Being taller plants (3 to 25 m), bamboo floral emissions in fields are at elevated levels, and intense enough to attract insects and bees onto them.

Visibly, floral portions, fruits and seeds are emitting strong blue at UV 366 nm in all grasses, including bamboos (Fig. 1b–l, n, p). Pollen grains in bamboos (Fig. 1j), the main reward for insects and bees, were strongly blue-emitting in UV. Grass leaves showed significant red emissions (multiple bands) of chlorophyll (Fig. 2a, S1), which absorbs sunlight leading to photosynthesis. Fluorescence from grass leaf matrices at UV 366 nm are mixed visual hues of chlorophyll red and FA-based blue emissions. Though grass leaves showed comparable contents of FA, mixing of dominant red and blue emissions results in less intense blue hues on leaf matrices in UV (Fig. 1e, f, n). Chlorophyll contents are very low in grass floral portions, fruits and seeds (Fig. 2a). *O. sativa* (Fig 2a, t6), *O. travancorica* (Fig 2a, t8) and *B. pallida* spikelets (Fig 2a, t10) showed faint red chlorophyll emissions. While most floral portions were blue-emitting, stigmatic portions and anthers of *O. travancorica* (Fig. 1j, l) and *B. pallida* (Fig. 1p) were found emitting mild red fluorescence at UV 366 nm. Blue emissions from grass reproductive structures for the most part are not mixed with chlorophyll or other emissions. Red chlorophyll bands were clearly visible in extracts of dicot leaves of *C. nutans* and *S. nodiflora* (Fig. 1q, S1). These dicot leaf extracts showed only very weak FA bands (Fig. S1). At UV 366 nm, relative FA/chlorophyll contents in dicot leaves, grass leaves and grass reproductive structures are reflected in their fluorescence emissions as red (Fig. 1q, S1), red-blue mix (Fig. 1e, f, n) and blue (Fig. 1b–l, n, p), respectively (Fig. 2a).

Visual cues from grass reproductive structures (blue) and leaves (red-blue) in UV effectively reflect the dominance of FA *versus* chlorophyll based emissions (Fig. 1, 2a). Most insects, bees (*Apis*, *Halictus*, *Trigona*, *Braunsapis*, *Ceratina*)^4^,^5^ and seed predators have their three vision maxima around 340 nm (UV), 430 nm (blue) and 535 nm (green)^18^. But, UV-induced red chlorophyll emissions are at λ 655–695 nm^26^,^32^. Flower and fruit visitors, therefore, cannot see the red emissions (as red) from grass leaves, unless they possess extra red receptors^16^,^17^,^18^,^19^,^20^,^33^. Similarly, stigmatic portions in grasses, particularly in bamboos, have mild red emissions, and insect visitations were not observed on them^4^,^5^. But, blue emissions (420–460 nm) from grass reproductive structures (Fig. 1, 2a, b) are within the vision maxima (430 nm, blue) of insects, bees and seed predators^16^,^17^,^18^,^19^,^20^,^21^,^22^,^23^,^32^. Faint UV induced red-blue mix emissions from grass leaves (Fig. 1e, f, n) and absence of insect visitations on mild red emitting stigma of bamboo florets^3^,^4^,^5^,^6^ are indications that insects and predators are directed towards FA-based blue-emitting vital reproductive structures (Fig. 1j, l, m, o, p). Grass floral parts that appear thin and hyaline in daylight, especially edges of glumes, lemma, palea, showed strong blue emissions at UV 366 nm, proportional to their FA contents (Fig. 1o, p). Significantly, FA provides photoprotection to vital cellular components in grass reproductive structures by absorption of damaging UV radiation and emitting as harmless blue patterns^26^,^28^,^34^.

In conclusion, our study found floral parts, fruits and seeds of grasses very ‘attractive' in UV induced fluorescence emissions. We propose these FA-based blue emissions from grass reproductive structures as enticing visual cues to pollinators (bees, other insects), seed dispensers (birds) and predators (birds, rats). This study thus provides more evidence that insect pollination is possible in grasses. Pollen transfer studies could further confirm entomophily in grasses. These blue emissions could also act as signals attracting insect pests to grains of cereals and millets. Also, we showed that, FA-based emissions are playing a crucial role in plant-animal interactions. Our findings could also help redefining the functions of grass floral parts and better understanding their morphology. Future studies on signaling molecules^12^,^13^,^14^ and defense mechanisms^35^,^36^ in grasses could lead to the discovery of novel molecular or fluorescence-based pest, weed control measures.

## Methods

### Grasses, UV emissions

Floral parts, fruits, seeds and leaves of six cereals, *Oryza sativa* L. (rice), *O. rufipogon* Griff. (red rice), *Triticum aestivum* L. (wheat), *Zea mays* L. (maize), *Sorghum bicolor* (L.) Moench (sorghum, ‘cholam'), *Sorghum bicolor* (L.) Moench (sorghum, ‘kattucholam'), two millets, *Eleusine coracana* (L.) Gaertn. (ragi, finger millet), *Pennisetum glaucum* (L.) R. Br. (bajra), three bamboos, *Ochlandra travancorica* (Bedd.) Gamble, *Bambusa pallida* Munro, *Melocanna baccifera* (Roxb.) Kurz and two common fodder grasses, *Pennisetum polystachion* (L.) Schult., *Axonopus compressus* (Sw.) P. Beauv. were collected from six locations in south India, (i) Jawaharlal Nehru Tropical Botanic Garden and Research Institute Bambusetum, Palode, (ii) College of Agriculture, Vellayani, (iii) Cropping System Research Centre, Karamana, (iv) Peringamala, Thiruvananthapuram, (v) Meenakshipuram, Virudhunagar and (vi) Bilagi, Bagalkot (see Supplementary information). Grass flowers, fruits, seeds, their dissected parts and leaves were scanned in white light and UV 366 nm in a Reprostar 3 with cabinet cover (CAMAG, Muttenz, Switzerland). For comparison, leaves of two dicots, *Clinacanthus nutans* (Burm.f.) Lindau (*Acanthaceae*) and *Synedrella nodiflora* (L.) Gaertn. (*Compositae*) were also scanned in white light and UV 366 nm.

### Alkaline hydrolysis and extraction of blue fluorescent fractions from grasses, estimation of ferulic acid

Fresh grass specimens, *Z. mays* (leaves, male flowers, external bracts, internal bracts, floral parts 3 g - lemma/palea/lodicule, immature seeds), *O. sativa* (leaves, spikelets, immature seeds), *E. coracana* (mature seeds), *O. travancorica* (leaves, spikelets, floral parts 3 g - lemma 1.39 g/palea 0.39 g/staminal filaments 0.92 g/pollen 0.3 g) and *B. pallida* (leaves, spikelets, floral parts 3 g - lemma 1.35 g/palea 0.75 g/lodicules 0.0045 g/immature seeds 0.89 g) were separately subjected to alkaline hydrolysis^37^ and extraction of FA. Dry seeds (stored over a season) of *Z. mays*, *O. sativa*, *T. aestivum* and *B. pallida* were also subjected to similar procedures. Each grass specimen (3 g) in a 100 ml RB flask was hydrolyzed with 30 ml of 3 M NaOH for 3 h under reflux. Reaction mixture was cooled down to room temperature, transferred to 250 ml beaker and acidified to pH ~ 2.0 with HCl. This acidified mixture with liberated phenolic acids was extracted with diethyl ether (30 ml × 3). Pooled diethyl ether fraction (reduced to 60 ml) was then extracted with 5% NaHCO_3_ (30 ml × 3). Aqueous fraction was again acidified to pH ~ 2 with HCl, and the free phenolic acids were extracted with diethyl ether (45 ml × 3). Diethyl ether fractions were concentrated under vacuum, redissolved in 1:1 water:methanol (5 ml) and stored at 4°C for analysis. Similar extraction procedures were also carried out on fresh leaves (3 g each) of dicots, *C. nutans* and *S. nodiflora*. These extracts and standard FA were spotted on silica gel plates 60F-254 (20 × 10 cm, 0.2 mm thickness, E. Merck, Darmstadt, Germany) by HPTLC (CAMAG, Muttenz, Switzerland), plates were developed in 9:1 acetonitrile-water and viewed at UV 366 nm. FA contents in extracts of leaves and reproductive structures of *Z. mays*, *O. sativa*, *T. aestivum*, *O. travancorica* and *B. pallida* were estimated by HPTLC-densitometry under identical conditions and developed spots were scanned at λ 322 nm. Mobile phase for estimation was optimized to 9.5:0.5 acetonitrile-water. Percentage FA contents (% ± SD, n = 4, based on fresh or dry weights,) were calculated from peak areas, using the standard curve (y = 25111x + 107.8, R^2^ = 0.996) with a linear relationship in the range 0.05 to 0.7 μg.

### Isolation of ferulic acid from *Triticum aestivum* seeds

*T. aestivum* seed extract (15 mg) in 1:1 water:methanol (400 μl) was applied on a silica gel plate 60F-254 (20 × 10 cm, 0.2 mm thickness, E. Merck, Darmstadt, Germany) as a band in preparative mode using HPTLC (CAMAG, Muttenz, Switzerland) and the plate was developed in 9:1 acetonitrile-water. Blue band of FA was marked on the plate under UV 366 nm. This blue marked band was scrapped off, methanol was added to the powder, sonicated, filtered and filtrate was concentrated under vacuum. This fraction (6 mg) was subjected to silica gel (60–120 mesh) column chromatography and eluted with chloroform-methanol (100:0, 99:1, 98:2, 96:4, 95:5, 90:10%). Chloroform-methanol (90:10) fraction yielded pure FA (3 mg) that was confirmed by co-TLC with standard FA.

### Ultraviolet absorption, fluorescence emission spectra

UV absorption spectra of FA isolated from *T. aestivum* seed extract and standard FA (Sigma-Aldrich, Bengaluru, India) were recorded on TLC Scanner 3 (CAMAG, Muttenz, Switzerland) and UV-1650PC UV-Visible spectrophotometer (Shimadzu, Kyoto, Japan). Fluorescence emission spectra of *T. aestivum* seed extract and FAs (isolated and standard) dissolved in methanol were measured on a SPEX Fluorolog F112X spectrofluorimeter (Horiba Jobin, Edison, USA) at an excitation λ 366 nm (Fig. 2c).

## Author Contributions

S.B. and K.C.K. designed the research, wrote the manuscript; S.B., A.J.J. and B.G.^1^ conducted UV scans, chemical analyses; K.C.K. selected grass species, collected specimens, carried out field observations and dissection of floral parts with B.G.^2^ and S.L.

## Supplementary Material

Supplementary Information Supplementary information

## Figures and Tables

**Table 1:** Ferulic acid (FA) contents estimated in grass reproductive structures

Grass specimen	FA content (% ± SD, n = 4, fresh wt.)
*Zea mays*	
Leaves	0.1162 ± 0.0043
Male flowers	0.2721 ± 0.00
External bracts	0.2057 ± 0.0037
Internal bracts	0.3757 ± 0.0025
Lemma/palea/lodicule	0.1129 ± 0.0055
Immature seeds	0.0832 ± 0.0003
Mature seeds (stored)	0.0932 ± 0.0015+
*Oryza sativa*	
Leaves	0.2574 ± 0.00
Spikelets	0.3654 ± 0.0069
Immature seeds	0.2998 ± 0.00
Mature seeds (stored)	0.0155 ± 0.00+
*Triticum aestivum*	
Mature seeds (stored)	0.0304 ± 0.00+
*Ochlandra travancorica*	
Leaves	0.2883 ± 0.0052
Spikelets	0.1278 ± 0.00
Lemma/palea/staminal filaments/pollen	0.0796 ± 0.0032
*Bambusa pallida*	
Leaves	0.2270 ± 0.0004
Spikelets	0.1543 ± 0.0007
Mature seeds (stored)	0.0984 ± 0.0010+

^+^ (% ± SD, n = 4, dry wt.).
