# Supplementary material for: UV induced visual cues in grasses
Source: Sci Rep. 2013 Sep 24;3:2738. doi: 10.1038/srep02738 (PMC3781395; doi:10.1038/srep02738)
Supplement: Supplementary Information [file srep02738-s1.doc]

**UV induced visual cues in grasses**

Sabulal Baby^1^*^±^, Anil John Johnson^1^, Balaji Govindan^1^, Sujith Lukose^2^, Bhaskaran Gopakumar^2^ & Konnath Chacko Koshy^2±^

^1^Phytochemistry and Phytopharmacology Division, Jawaharlal Nehru Tropical Botanic Garden and Research Institute, Pacha-Palode, Thiruvananthapuram 695562, Kerala, India. ^2^Plant Genetic Resources Division, Jawaharlal Nehru Tropical Botanic Garden and Research Institute, Pacha-Palode, Thiruvananthapuram 695562, Kerala, India.

**Supplementary information**

# Voucher specimens of grasses (monocots, *Poaceae*), dicots and bees deposited at TBGT

**Cereals:** *Oryza* *sativa* L., 17.12.2012, *B. Gopakumar 64255*; 6.5.2013, *K. C. Koshy* *64524*; *Oryza* *rufipogon* Griff., 20.12.2012, *B. Gopakumar 70538*; *Sorghum* *bicolor* (L.) Moench, 22.1.2013, *V. B. Hosagoudar 64516*; *Sorghum* *bicolor* (L.) Moench (fodder plant), 11.1.2013, *G. Balaji 64511*; *Triticum* *aestivum* L., 23.1.2013*, K. C. Koshy 64513*; 17.6.2013, *V. B. Hosagoudar 64523*; *Zea* *mays* L., 10.1.2013, *K. C. Koshy 64509*; 5.6.2013, *K. C. Koshy 64522.*

**Millets:** *Eleusine coracana* (L.) Gaertn., 23.01.2013, *K. C. Koshy 64514*; *Pennisetum* *glaucum* (L.) R. Br., 10.01.2013, *B. Sabulal 64510.*

**Bamboos:** *Bambusa* *pallida* Munro, 3.01.2013, *Sujith Lukose 70540*; *Melocanna* *baccifera* (Roxb.) Kurz, 18.12.2012, *K. C. Koshy 64508*; *Ochlandra* *travancorica* (Bedd.) Gamble, 19.12.2012, *Sujith Lukose 70544.*

**Common grasses:** *Axonopus* *compressus* (Sw.) P. Beauv., 27.2.2013, *K. C. Koshy 70542*; *Pennisetum polystachion* (L.) Schult., 27. 2. 2013, *K. C. Koshy 70543.*

**Dicots:** *Clinacanthus* *nutans* (Burm.f.) Lindau (*Acanthaceae*), 27.2.2013, *K. C. Koshy 70545*; *Synedrella* *nodiflora* (L.) Gaertn. (*Compositae*), 27.2.2013, *Sujith Lukose 70544.*

**Bees:** *Apis cerana indica* Fabricus ([*Apidae*](http://en.wikipedia.org/wiki/Apidae)), 7.7.2003, *B. Gopakumar 6714-01*; *Apis dorsata* Fabricus ([*Apidae*](http://en.wikipedia.org/wiki/Apidae)), 28.4.1999, *s. coll. 281-01*; *9.5.2010*, *B. Gopakumar 66837*; *Halictus taprobane* Cameron ([*Halictidae*](http://www.discoverlife.org/mp/20q?search=Halictidae)), 7.7.2003, *B. Gopakumar 6714-02*; 1.7.2003, *B. Gopakumar 6719-01*; *Trigona irridipennis* Smith ([*Apidae*](http://en.wikipedia.org/wiki/Apidae)), *B. Gopakumar 6414-04.*

**Fig. S1**


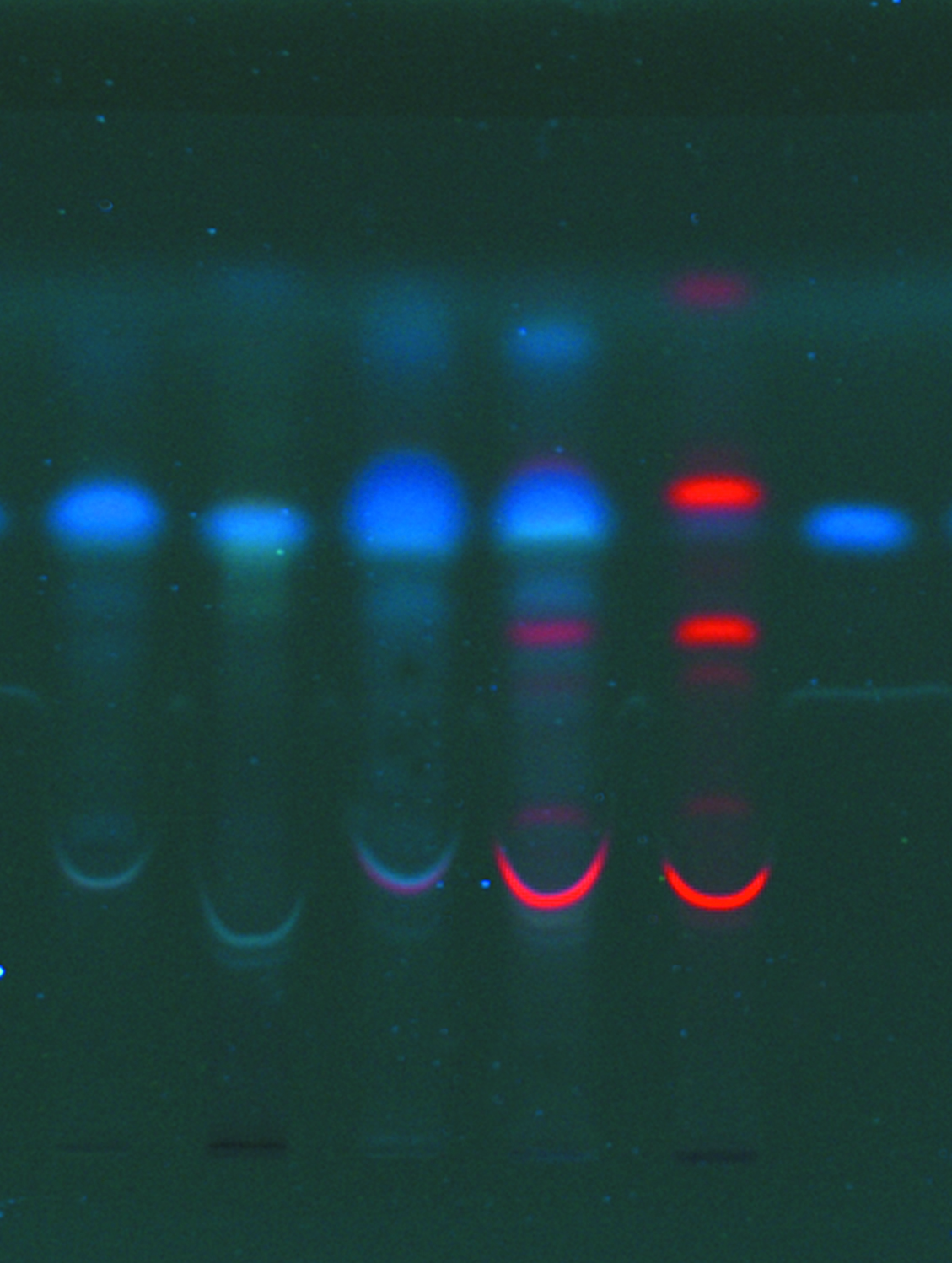


**Figure S1 HPTLC profiles of blue metabolites isolated from seeds, floral parts, leaves of grasses and dicot leaves at UV 366 nm.** Tracks (left to right) (t1) *Triticum aestivum* seeds, (t2) *Elucine coracana* seeds, (t3) *Bambusa pallida* floral parts, (t4) *B. pallida* leaves, (t5) *Clinacanthus nutans* leaves and (t6) standard FA.
